# Flavonoids as Molecules With Anti-*Zika virus* Activity

**DOI:** 10.3389/fmicb.2021.710359

**Published:** 2021-09-10

**Authors:** Allan Henrique Depieri Cataneo, Eloah Pereira Ávila, Larissa Albuquerque de Oliveira Mendes, Viviane Guedes de Oliveira, Camila Rodrigues Ferraz, Mauro Vieira de Almeida, Sandra Frabasile, Claudia Nunes Duarte dos Santos, Waldiceu Aparecido Verri, Juliano Bordignon, Pryscilla Fanini Wowk

**Affiliations:** ^1^Laboratório de Virologia Molecular, Instituto Carlos Chagas/Fiocruz-PR, Curitiba, Brazil; ^2^Departamento de Química, Universidade Federal de Juiz de Fora, Juiz de Fora, Brazil; ^3^Departamento de Ciências Patológicas, Centro de Ciências Biológicas, Universidade Estadual de Londrina, Londrina, Brazil; ^4^Sección Virologia, Facultad de Ciencias, Universidad de La República, Montevideo, Uruguay

**Keywords:** antiviral, flavonoid, natural products, Zika treatment, Zika virus

## Abstract

*Zika virus* (ZIKV) is an arthropod-born virus that is mainly transmitted to humans by mosquitoes of the genus *Aedes* spp. Since its first isolation in 1947, only a few human cases had been described until large outbreaks occurred on Yap Island (2007), French Polynesia (2013), and Brazil (2015). Most ZIKV-infected individuals are asymptomatic or present with a self-limiting disease and nonspecific symptoms such as fever, myalgia, and headache. However, in French Polynesia and Brazil, ZIKV outbreaks led to the diagnosis of congenital malformations and microcephaly in newborns and Guillain-Barré syndrome (GBS) in adults. These new clinical presentations raised concern from public health authorities and highlighted the need for anti-Zika treatments and vaccines to control the neurological damage caused by the virus. Despite many efforts in the search for an effective treatment, neither vaccines nor antiviral drugs have become available to control ZIKV infection and/or replication. Flavonoids, a class of natural compounds that are well-known for possessing several biological properties, have shown activity against different viruses. Additionally, the use of flavonoids in some countries as food supplements indicates that these molecules are nontoxic to humans. Thus, here, we summarize knowledge on the use of flavonoids as a source of anti-ZIKV molecules and discuss the gaps and challenges in this area before these compounds can be considered for further preclinical and clinical trials.

## Introduction

*Zika virus* (ZIKV) is an arthropod-born virus belonging to the family *Flaviviridae* and genus *Flavivirus*. The first isolation of ZIKV occurred in 1947 from a sentinel rhesus monkey serum sample from the Zika forest in Uganda, when a surveillance program for yellow fever was in progress (Dick et al., 1952).

Since its first isolation in the 1940s, human ZIKV infections have occurred mainly across Asia and Africa and are often associated with mild clinical manifestations. Most ZIKV infections in humans are asymptomatic, while 20–25% of infections develop into a self-limiting mild illness with flu-like symptoms, such as fever, myalgia, headache, maculopapular rash, and lower back pain (Calvet et al., 2016).

Until 2007, only a few cases of ZIKV infection had been reported worldwide; however, during that year, an outbreak was reported on Yap Island, Federated States of Micronesia, with an estimated 5,005 infections among the 6,892 residents (Duffy et al., 2009). In October 2013, ZIKV infection was confirmed in French Polynesia and led to a massive outbreak (Cao-Lormeau et al., 2014). Before the outbreak in French Polynesia, there were no reports of severe disease related to ZIKV infection; however, the 2013 French Polynesia outbreak consisted of several patients presenting neurological symptoms, including Guillain-Barré syndrome (GBS), which was later associated with ZIKV infection (Oehler et al., 2014). Early in 2015, ZIKV autochthonous transmission was reported in the Northern region of Brazil (Zanluca et al., 2015).

**Figure 1:**
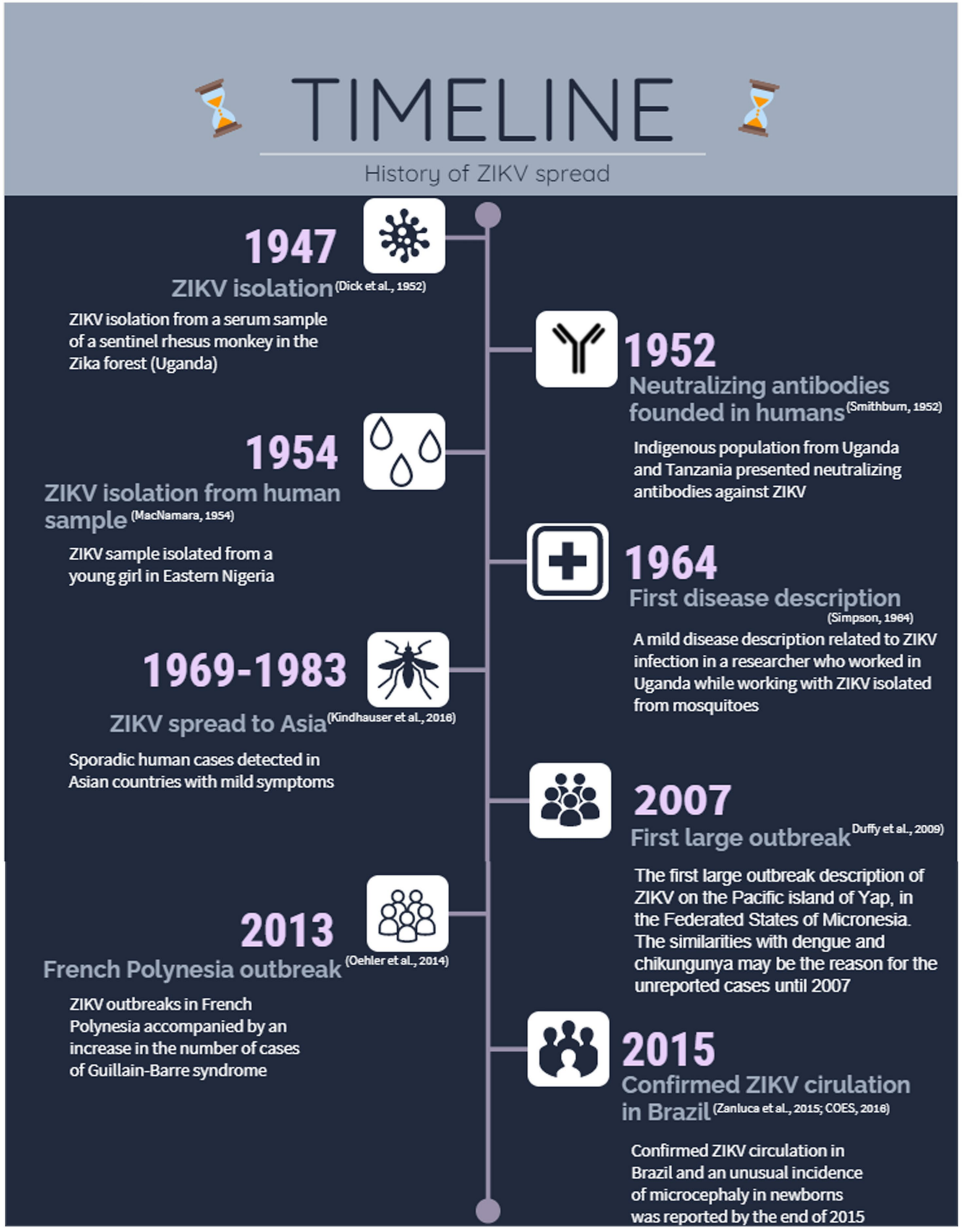
*Zika virus* (ZIKV) spread since its isolation until the outbreak in Brazil in 2015. Representative timeline infographic highlighting the main events related to the spread of ZIKV.

Since the first detection of ZIKV circulation in Brazil in 2015, more than 240,000 cases have been reported (Ministério da Saúde, 2019a), and approximately 3,000 cases of microcephaly and other congenital malformations associated with ZIKV infection in newborns have been confirmed (Ministério da Saúde, 2019b). Children with microcephaly present with health disorders, such as seizures and brain and eye damage, which restrict their development and have both health and social impacts. Therefore, the rapid spread of this emerging arbovirus and recent evidence of severe nervous system disease have attracted the attention of the scientific community. The infographic timeline in Figure 1 summarizes the most important landmarks of ZIKV since its initial detection.

Thus, based on the impact of the ZIKV epidemic and congenital syndrome observed in newborns, it is of utmost importance to pursue research on strategies to prevent and treat infected patients, including vaccines and antiviral strategies to avoid or reduce the damage caused by the infection.

## ZIKV Biology

*Zika virus* has been demonstrated to infect a variety of human cells, such as skin fibroblasts, neural progenitor cells, monocytes, dendritic cells, and Hofbauer cells (Hamel et al., 2015; Quicke et al., 2016; Tang et al., 2016; Bowen et al., 2017; Michlmayr et al., 2017). C-type lectin receptors such as DC-SIGN and transmembrane phosphatidylserine receptors such as TIM (TIM1, TIM3, and TIM4) and TAM (TYRO3, AXL, and MER) seem to mediate ZIKV entry into target cells (Hamel et al., 2015). After cell attachment, *Flavivirus* entry occurs through clathrin-mediated endocytosis followed by endosome acidification and exposure of the fusion loop of the surface protein that allows fusion between the viral envelope and endosomal membrane; finally, the viral RNA is released into the cell cytoplasm (Modis et al., 2004).

The ZIKV genome is a positive single strand of RNA with approximately 11,000 bases encoding a polyprotein, which is processed by host and viral proteases into three structural proteins, envelope (E), premembrane/membrane (prM/M), and capsid (C), and seven nonstructural proteins (NS1, NS2A, NS2B, NS3, NS4A, NS4B, and NS5; Kuno and Chang, 2007).

The flavivirus E protein allows viral particles to bind to cell receptors and is an important target for neutralizing antibodies (Dai et al., 2016). The C protein is mainly associated with virus assembly by packing of the RNA genome; moreover, it has been suggested that this protein may play a role in the fusion process (Freire et al., 2015). The prM/M protein is essential in the late steps of the viral replication cycle, and the cleavage of prM and M at this step plays a central role in virus maturation and the effective production of infective particles. Moreover, prM amino acid mutations have been suggested to be involved in the emergence of fetal microcephaly associated with ZIKV infection (Li et al., 2008; Yuan et al., 2017).

Nonstructural (NS) proteins are essential in the *Flavivirus* replication cycle as well as in immune response evasion and pathogenesis. The *Flavivirus* NS1 protein has been demonstrated to play an important role in helping viral replication and is associated with pathogenesis by increasing vascular permeability (Youn et al., 2012; Puerta-Guardo et al., 2019). NS2B is the cofactor for the NS3 protease; therefore, the NS2B-NS3 complex is essential for optimal catalytic activity and cleavage of specific regions in the precursor polyprotein. NS3 is a bipartite protein with both a protease and helicase domain. This protein plays an important role in cleaving the polyprotein and unwinding the RNA secondary structure to allow the synthesis of viral RNA copies (Bollati et al., 2010). NS4A and NS4B, together with NS2A and NS2B, act as scaffolds for the replication complex that is essential to viral replication (Zou et al., 2015). The NS5 protein is an RNA-dependent RNA polymerase that is necessary for RNA replication and has a methyltransferase domain, which is required for the RNA capping process (Egloff et al., 2007). Additionally, NS proteins have been described to have the ability to circumvent the immune response, which is critical for viral replication success. *Flaviviridae* NS proteins have been suggested to inhibit RIG-I signaling (Dalrymple et al., 2015) and suppress the type I interferon response (Grant et al., 2016) as well NF-κB activation (Chen et al., 2015). Altogether, these viral proteins play fundamental roles during infection, viral replication, and immune response evasion; therefore, they are relevant targets for antiviral compound design (Mottin et al., 2018a).

## Drug Discovery Research Targeting ZIKV

There have been successful strategies that support drug discovery against emerging viruses, such as drug repurposing, computational-based drug discovery, and the search for natural products (Teixeira et al., 2014; da Silveira Oliveira et al., 2017; Sacramento et al., 2017; Mottin et al., 2018b).

Drug repurposing is a strategy that finds new uses for a previously approved drug. This is an attractive strategy since the drug has already been approved in terms of safety for human use. As an example, the FDA-approved drugs chloroquine, an anti-malaria drug, and sofosbuvir, an antiviral drug used for hepatitis C virus treatment, both exert anti-ZIKV activity (Sacramento et al., 2017).

Additionally, an example of a computational-based technique used in drug discovery against ZIKV is the OpenZika project, in which *in silico* analysis searches for anti-ZIKV drugs among millions of compounds (Ekins et al., 2016). The OpenZika platform uses compounds from the ZINC database, FDA-approved drugs, and the National Institutes of Health (NIH) clinical collection in collaboration with the IBM World Community Grid (WCG)^1^ to virtually screen millions of compounds against crystal structures of ZIKV proteins or related viruses.

In addition, the search for natural product-derived compounds against pathogens has attracted the attention of researchers in the drug discovery field in recent decades. Natural products have been used to identify new molecules to find antiviral drugs (Ninfali et al., 2020).

### Flavonoids: Biosynthesis, Structures, and Sources

Natural products and their derivatives are an important source of bioactive compounds and represent more than one-third of all FDA-approved new molecules (Patridge et al., 2016). Among natural products, flavonoids are one of the classes of major secondary metabolites that occur naturally and ubiquitously in plants (Yonekura-Sakakibara et al., 2019). Flavonoids can be found as glycoside conjugates, therefore, contributing to the complexity of this class of metabolites, as over 6,000 flavonoids have been identified (Beecher, 2003; Kozłowska and Szostak-Węgierek, 2018). The structure of these polyphenolic compounds is, in general, broadly characterized by a skeleton structure containing 15 carbons, a heterocyclic ring (ring C), and two phenyl rings (rings A and B).

Flavonoid biosynthesis begins with the condensation of one molecule of p-coumaryl-CoA derived from chiquimate (ring B), with three molecules of malonyl-CoA of polyketide origin (ring A) mediated by the enzyme chalcone synthase (CHS), giving rise to a chalcone. Flavanone isomerization occurs *via* the enzyme flavanone isomerase (CHI). The pathway then separates into lateral branches under the action of different enzymes, such as isomerases, hydroxylases, and reductases, leading to different classes of flavonoids (Santos et al., 2017).

**Figure 2:**
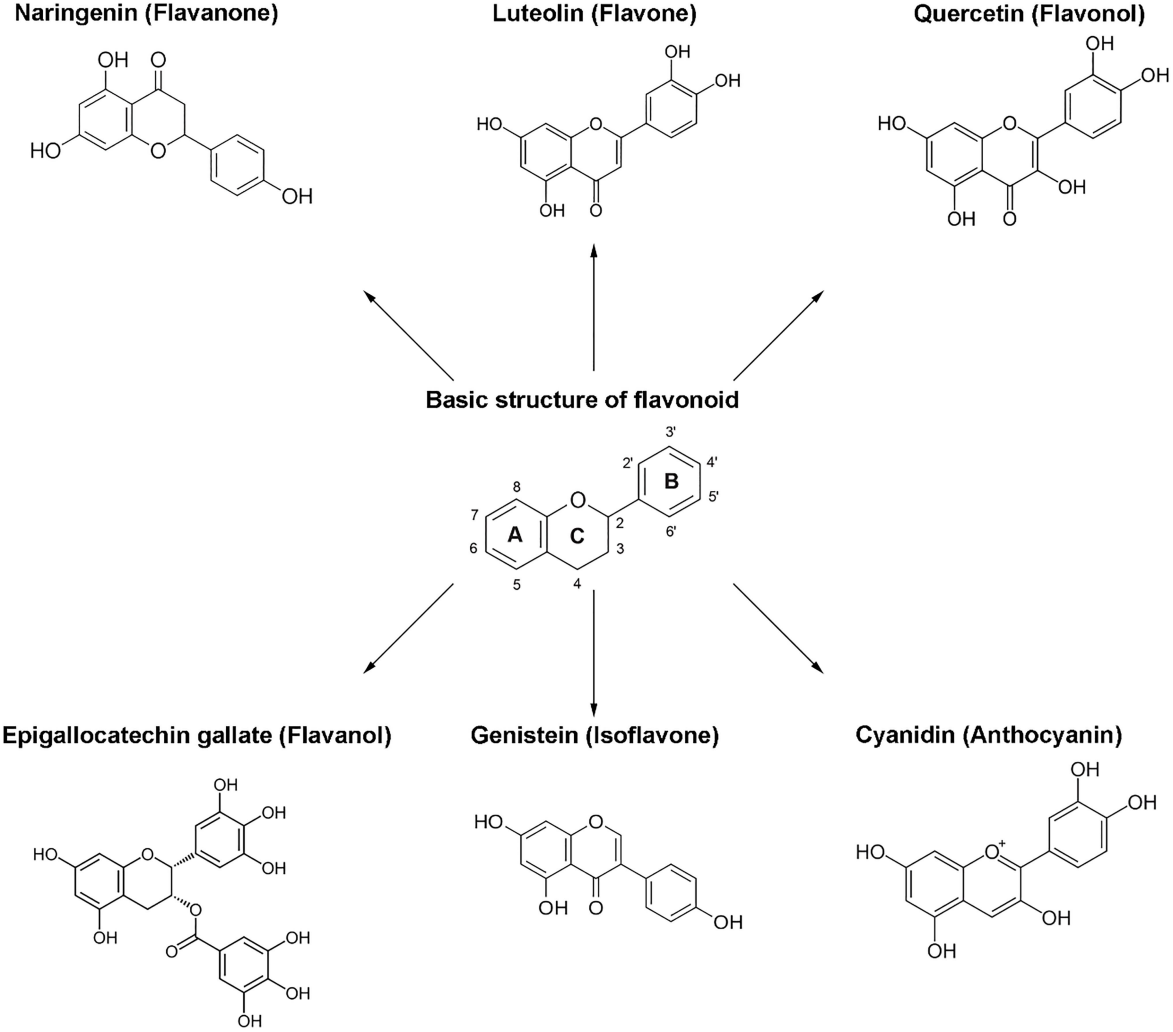
Chemical structures of flavonoids and their subclasses. The basic flavonoid structure is presented in the center. The arrows illustrate examples of compounds for each flavonoid subclass.

The classification of flavonoids into subgroups is generally based on modifications of the C ring, whereas within the same group, the classification is based on modifications to the A and B rings. Moreover, some carbons of the skeleton structure can be replaced with hydroxyl, methoxyl, and other groups (Kozłowska and Szostak-Węgierek, 2018). Therefore, flavonoids can be classified into six major groups: flavonols, flavanones, flavones, flavanols, isoflavones, and anthocyanins (Figure 2). Flavones, flavonols, and isoflavones are characterized by a planar structure due to the presence of a double bond in the central ring (Kumar and Pandey, 2013). Flavanones and flavanols present at least one stereogenic center with a tetrahedral geometry due to the presence of two sp3 carbons.

Flavonoids are widely encountered in dietary sources, mainly vegetables, fruits, seed roots, and cereals (Kozłowska and Szostak-Węgierek, 2018). Despite being widely distributed among different sources, the presence of flavonoids in plants may vary depending on numerous factors, such as soil composition, climate, season, storage conditions, and plant species (Patil et al., 1995; Van der Sluis et al., 2001). In addition, food processing, such as cooking, may result in flavonoid transformation or losses of their content (Beecher, 2003).

### Biological Properties of Flavonoids

The biological/pharmacological activity of flavonoids was first revealed almost 100years ago in 1936. It was demonstrated that ascorbic acid was not effective in the treatment of purpura unless it was used in combination with extracts of Hungarian red pepper or lemon juice, which restored normal vascular permeability. The authors fractionated the extracts and described the active molecule as substance P due to its action on vascular permeability. Later, substance P was called citrin, and it was demonstrated that hesperidin, a flavonoid, was its major component (Bruckner and Szent-Györgyi, 1936; Rusznyák and Szent-Györgyi, 1936). Currently, it is well established that flavonoids present diverse biological/pharmacological activities, such as antioxidant, antimicrobial, antitumor, and immunomodulatory activities, which make this class of molecules a potential source of new compounds for industry and medicine.

The antioxidant mechanisms of flavonoids include (i) quenching free radical components, (ii) chelating metals, (iii) inhibiting the activity of enzymes associated with the generation of free radicals, and (iv) inducing the expression of endogenous antioxidant enzymes (Kumar and Pandey, 2013). Of note, flavonoids may also act as prooxidants according to their concentration, structure, and hydroxyl substitutions (Cao et al., 1997).

Flavonoids can modulate the immune response through the induction of cytokines and proinflammatory gene expression (Nadella et al., 2019). Luteolin, quercetin, and rutin seem to promote macrophage polarization toward the M2 phenotype. Depending on the disease context, this macrophage phenotype plays a role in tissue repair and limiting inflammation (Nadella et al., 2019; Wang et al., 2019). Quercetin (Guazelli et al., 2018), apigenin (Cardenas et al., 2016), diosmin (Fattori et al., 2020), rutin (Carvalho et al., 2019), and hesperidin methyl chalcone (Rasquel-Oliveira et al., 2020) inhibit the activation of NF-κB, an important inflammatory transcription factor involved in a variety of diseases. Flavonoids such as quercetin (Guazelli et al., 2018) and vitexin (Nikfarjam et al., 2017) reduce proinflammatory cytokine production. Quercetin (Lee et al., 2018), vitexin (Jiang et al., 2019), and naringenin (NAR; Zhang et al., 2019) inhibit MAP kinase signaling cascades in macrophages, and quercetin (Domiciano et al., 2017) inhibits NLRP3 inflammasome activation. Apigenin inhibits COX-2, the cytokines IL-1β, IL-2, IL-6, IL-8, and TNF-α, iNOS, and AP-1 production in the context of LPS cellular activation (Patil et al., 2016).

Flavonoids have the potential for the treatment of some types of cancer by modulating the cell cycle, heat-shock proteins, tyrosine kinases, p53 proteins, and Ras proteins (Chahar et al., 2011). Quercetin reduces metastasis by inhibiting migration and invasion pathways in gastric cancer cell models (Li and Chen, 2018). Moreover, the flavanone hesperetin induces apoptosis in gastric cancer cells by producing reactive oxygen species, promoting changes in the mitochondrial membrane potential, and decreasing the antiapoptotic/proapoptotic (Bcl-2/Bax) protein ratio (Zhang et al., 2015). Thus, flavonoids may represent a potential source for future anticancer drugs.

Additionally, due to the increase in antibiotic resistance, there is a special need to find and develop new antibacterial agents, and flavonoids also represent a potential source of candidate molecules. *In vitro*, apigenin and luteolin are active against methicillin-resistant *Staphylococcus aureus* (MRSA) and methicillin-sensitive *S. aureus* (MSSA; Sato et al., 2000). Additionally, galangin purified from a propolis ethanol extract shows bactericidal activity against MRSA, *Enterococcus* spp., and *Pseudomonas aeruginosa in vitro* (Pepeljnjak and Kosalec, 2004). The extract of the leaves of *Oncoba spinosa* Forssk (Salicaceae), quercetin, apigenin-7-O-β-D-glucuronopyranoside, quercetin 3-O-β-D-galactopyranoside, and quercetin 3-O-α-L-rhamnopyranosyl (1→6) β-D-glucopyranoside have demonstrated antimicrobial effects against *Enterobacter aerogenes*, *Escherichia coli*, *Klebsiella pneumoniae*, and *S. aureus* (Djouossi et al., 2015). Therefore, there is potential for the use of flavonoids as antibacterial drugs, although *in vivo* validation is still required.

The antiviral activity of flavonoids was first described by Béládi et al. (1965). The authors showed the possible virucidal effects of quercetin and morin against herpesvirus (Béládi et al., 1965). A few years later, the same authors demonstrated the antiviral effects of quercetin, rutin, morin, luteolin, apigenin, and fisetin on herpes simplex virus and parainfluenza virus type 3 (Béládi et al., 1977). The data suggested that the antiviral activity was associated with the flavonoid molecular structure, and it was postulated that the number and position of hydroxyl groups and glycosides could modulate the antiviral activity. This type of structure probably interferes with virus binding to their targets, modulating electrostatic interactions such as hydrogen bonds and ionic interactions (Béládi et al., 1977; Liu et al., 2008). Since then, numerous studies have evaluated the antiviral activity of flavonoids against several viruses. Quercetin and morin provide protection to mice infected with Mengo virus, a member of the *Picornaviridae* family (Veckenstedt et al., 1978). Quercetin, hesperetin, catechin, and naringin were tested against herpes simplex virus type 1, parainfluenza virus type 3, poliovirus type 1, and respiratory syncytial virus with promising results, except for naringin, which did not show any antiviral activity (Kaul et al., 1985). Some flavonoids have also been described as inhibitors of HIV infection (Mahmood et al., 1993). Quercetin-3b-galactoside binds to the SARS-CoV 3CL protease, inhibiting its proteolytic activity (Yi et al., 2004). Since SARS-CoV-2 protease 3CL maintains the same Gln189 site (Zhang et al., 2020), where quercetin binds to SARS-CoV 3CLpro (Chen et al., 2006), quercetin may represent a potential approach to prevent and treat COVID-19 (Colunga Biancatelli et al., 2020).

The effects of flavonoids against arboviruses of medical importance have been extensively studied in the past few years, mainly due to the emergence and reemergence of arboviruses. *Dengue virus* (DENV) studies have suggested that this class of compounds could impact viral replication (Zandi et al., 2011; de Sousa et al., 2015; Frabasile et al., 2017). Furthermore, the flavonoids apigenin, chrysin, NAR, and silybin seem to impair the viral entry and replication of the *Chikungunya virus* (CHIKV) and *Semliki Forest virus* in BHK cells (baby hamster kidney fibroblasts; Pohjala et al., 2011). Recently, Murali et al. (2015) demonstrated the anti-CHIKV effects of the ethanolic extract of *Cynodon dactylon*. By reversed-phase HPLC and GC-MS, they observed that the major constituents of the ethanolic extracts are luteolin and apigenin (Murali et al., 2015).

### Anti-ZIKV Activity of Flavonoids

Despite the reduction in the incidence of ZIKV infection in Brazil, from 200,000 suspected cases in 2016 to 9,000 cases in 2019, ZIKV continues to circulate and affect human health in South America. Moreover, nonhuman primates may be involved in the maintenance of ZIKV transmission and circulation (Terzian et al., 2018). During 2018, changes in the growth and development of 3,332 children were related to ZIKV or other infectious etiologies. Between January and August 2019, two deaths due to ZIKV infection and 447 cases of pregnant women becoming infected with ZIKV were confirmed by the Brazilian Ministry of Health (Ministério da Saúde, 2019b). During the first semester of 2020, 5,959 cases around the country were reported (Ministério da Saúde, 2020). Despite the global effort to develop specific anti-ZIKV drugs and a vaccine, only one vaccine has reached phase 2 clinical trials (U.S. National Institutes of Health, 2019).

**Figure 3:**
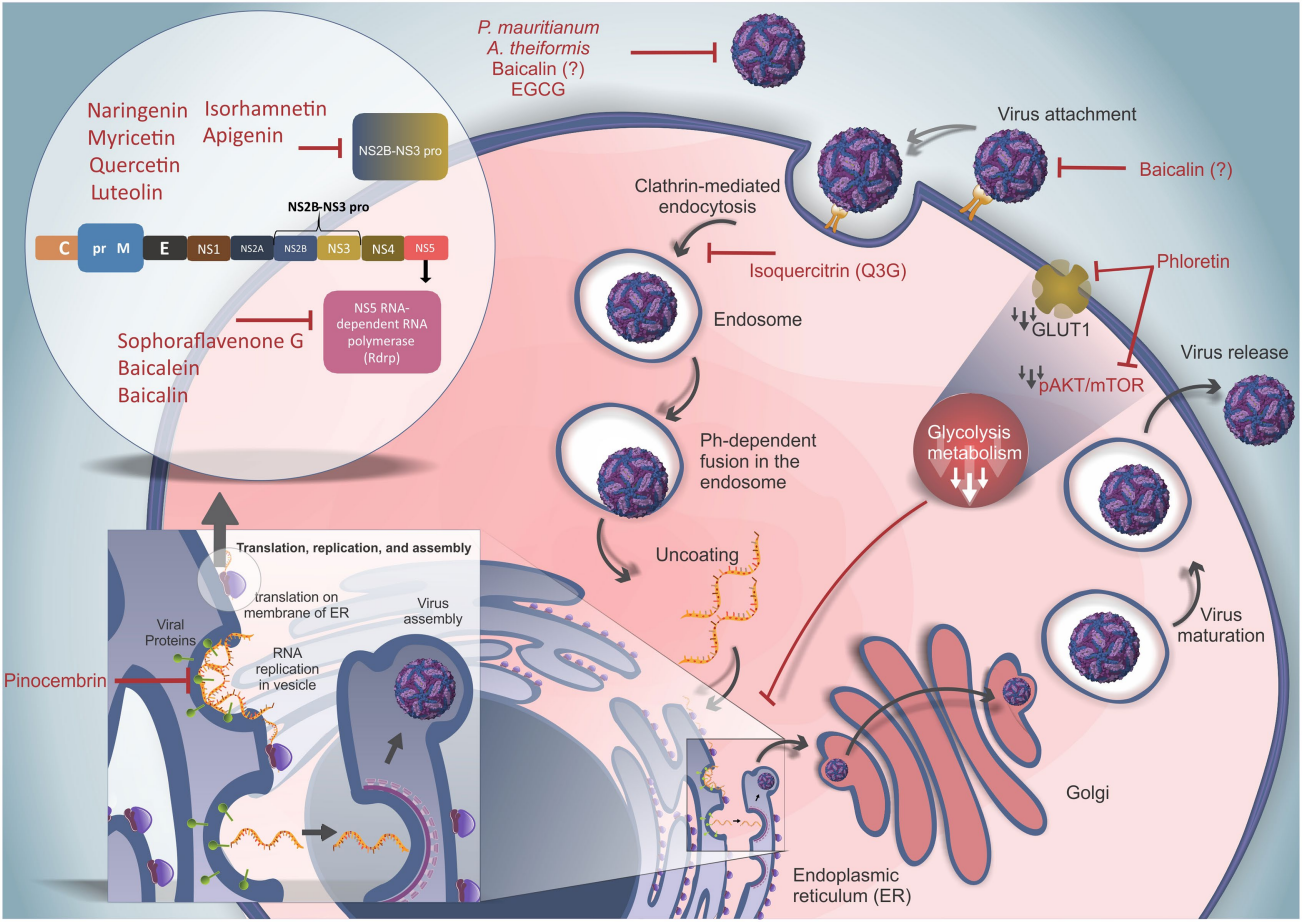
Replication cycle of ZIKV and the actions of different flavonoids. Free viral particles can be disrupted by the actions of curcumin, *Psiloxylon mauritianum* extract, *Aphloia theiformis* extract, baicalin, and epigallocatechin gallate (EGCG) before binding of the virus to cell receptors. ZIKV attaches to host receptors and is internalized by clathrin-mediated endocytosis. This phase can be inhibited by curcumin and baicalin, but the inhibitory mechanism needs to be clarified. Isoquercitrin (Q3G) inhibits the internalization and trafficking of viral particles. The acidic microenvironment within endosomes allows the fusion between viral and endosomal membranes, resulting in viral RNA release into the cytoplasm. The viral RNA is translated into a polyprotein (inset in the upper left corner shows the sequential and structural organization of the ZIKV polyprotein) containing three structural proteins [capsid (C), premembrane (prM), and envelope (E)] and seven nonstructural proteins (NS1, NS2A, NS2B, NS3, NS4A, NS4B, and NS5). The viral polyprotein is cleaved by host and viral proteases. NAR, myricetin, quercetin, luteolin, isorhamnetin, apigenin, and curcumin can inhibit the NS2B-NS3 protease (NS2B-NS3 pro). Conversely, sophoraflavenone G, baicalein, and baicalin can inhibit NS5-RNA polymerase (RDRP). Moreover, the NS3 protein interacts with RDRP to allow viral genome replication, and pinocembrin can impair viral RNA synthesis. Phloretin was demonstrated to decrease glycolytic metabolism by downregulating the pAKT/mTOR pathway and GLUT1 blockade, impairing ZIKV replication by downmodulating energetic metabolism. (?) Highlights molecules with inconsistent results.

Therefore, the screening of the anti-ZIKV activity of natural flavonoids has been an important area of investigation due to the biological effects already described for these molecules. Recent advances in research on flavonoid-based anti-ZIKV molecules and the current understanding of their mechanisms of action are summarized below and depicted in Table 1 and Figure 3.

**Table 1:** *In vitro* activity of flavonoids against ZIKV.

Compound	Chemical structure	Proposed antiviral mechanism	References
Epigallocatechin gallate (EGCG)	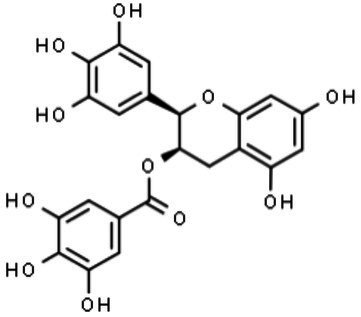	Blockage of viral-host membrane fusion	Carneiro et al., (2016), Sharma et al., (2017)
Quercetin-3-β-O-D-glucoside (Q3G)	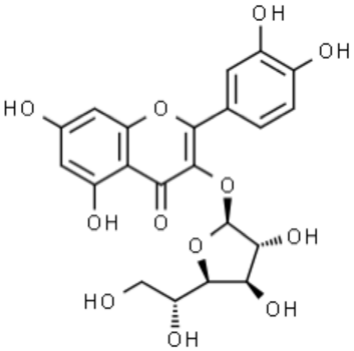	Inhibition of internalization and trafficking of viral particles	Wong et al., (2017), Gaudry et al., (2018)
Sophoraflavenone G	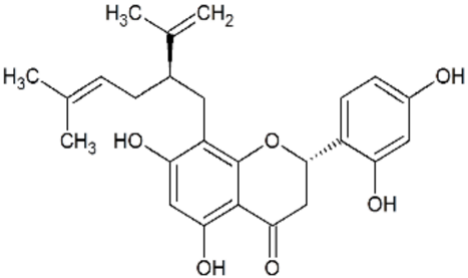	Inhibition of viral RNA-dependent RNA polymerase (RdRP)	Sze et al., (2017)
Baicalein	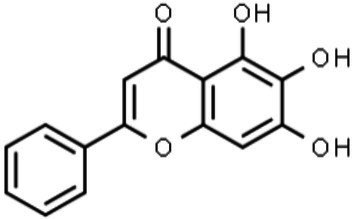	Unknown; docking analysis suggests NS5 binding	Oo et al., (2019)
Baicalin	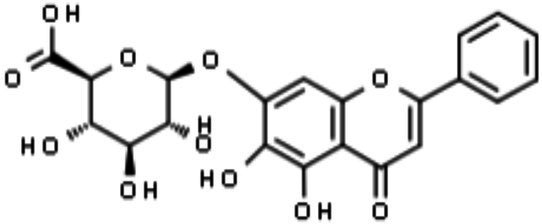	Unknown; docking analysis suggests NS5 binding	Oo et al., (2019)
Phloretin	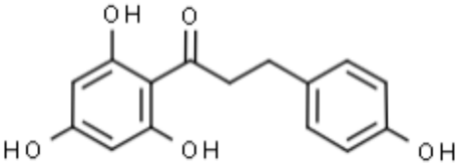	Interference with glucose metabolism required for ZIKV replication	Lin et al., (2019)
Pinocembrin	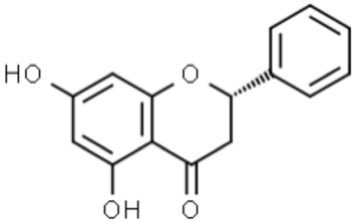	Inhibition of viral RNA and envelope protein synthesis	Lee et al., (2019)
Naringenin	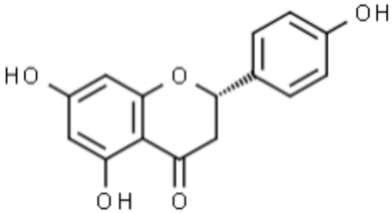	Unknown; docking analysis suggests NS2B-NS3 protease binding	Cataneo et al., (2019)
Quercetin	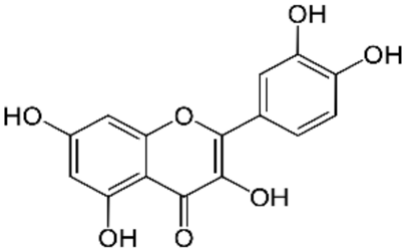	Non-effective	Gaudry et al., (2018)
Hyperoside	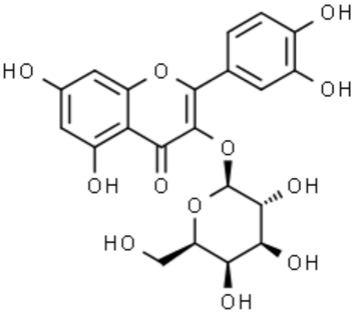	Non-effective	Gaudry et al., (2018)
Kaempferol	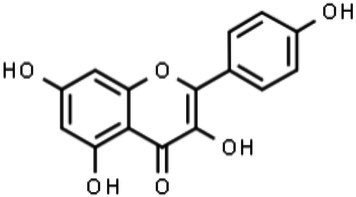	Non-effective	Gaudry et al., (2018)

Source of chemical structures: https://www.chemspider.com/Default.aspx.

#### *In silico* Prediction of Anti-ZIKV Drugs

*In vitro* and *in vivo* screening of drug candidates or libraries of compounds has been the predominant process for antiviral drug discovery. Recently, the use of computational-based strategies to rationally select compounds has increased in the field of drug discovery. The *in silico* approach may reduce the number of candidate compounds to be tested *in vitro* and *in vivo*, as this approach works as a filter to screen molecules and predict molecular efficacy (Terstappen and Reggiani, 2001). Thus, computational-based strategies applied in drug discovery have the potential to reduce the costs of *in vitro* and *in vivo* assays and the time until a new drug reaches the market. However, despite optimizing the research for new antiviral drugs, computational-based strategies require the determination of viral proteins’ structures together with effective computational power and scientific expertise in selecting potential *hits*.

One example of the use of computational-based strategies to find drugs against ZIKV was the OpenZika project (Ekins et al., 2016). The plan was to run dock experiments between ZIKV protein structures and millions of compounds to select potential hits using an IBM World Community Grid Project. Thus, structural protein determination and nucleic acid conformation together with docking analysis and modeling studies are extremely important for the discovery and development of new drug candidates against emerging viruses (Saw et al., 2017).

In this context, flavonoids have been used in docking analyses to determine potential interactions with viral proteins. The compound epigallocatechin gallate (EGCG) was demonstrated by a docking strategy to impair the fusion of ZIKV with the cellular membrane (Sharma et al., 2017). Enzyme inhibition kinetic assays and docking studies identified a set of natural flavonoids as potential inhibitors of DENV NS2B-NS3, which are essential nonstructural proteins involved in the replication complex (de Sousa et al., 2015). Given the similarities between DENV and ZIKV, it is plausible to assume that these same flavonoids would also inhibit ZIKV proteases. Indeed, a study by Roy et al. (2017) on the structural characterization of ZIKV NS2B-NS3 protease identified five compounds (myricetin, quercetin, luteolin, isorhamnetin, and apigenin) that act as inhibitors of viral protease (Roy et al., 2017). Computational approaches have been useful in drug discovery studies by helping to select hits and better guide the *in vitro* and *in vivo* validation of candidates.

#### Flavonoids Extracted From Plants With Anti-ZIKV Potential

Considering the potential antiviral properties of flavonoids and the high contents of these natural compounds in *Psiloxylon mauritianum*, its anti-ZIKV activity was tested. In a time-course experiment, addition of the *P. mauritianum* polyphenolic aqueous extract showed effectiveness only during the initial steps of viral infection. Binding- and internalization-specific assays displayed a 10-fold inhibition in ZIKV-infected Vero cells compared with the untreated control. Moreover, the results showed that preincubation of the virus with the extract reduced infectivity by ~80% (Clain et al., 2019). Despite the promising results, the polyphenol extract derived from *P. mauritianum* is composed of several flavonoids, and the exact active compound remains to be determined.

Another aqueous plant extract derived from the aerial parts of *Aphloia theiformis* also showed antiviral activity against ZIKV, inhibiting the early steps in viral infection. Phytochemical analysis demonstrated that the *A. theiformis* extract was rich in polyphenol content (Clain et al., 2018), but the active compound(s) against ZIKV in the extract are unknown. Testing fractionated compounds from an active extract may provide key answers to understand whether the compounds are effective alone or act synergistically with other molecules.

A study carried out with the hydroalcoholic extract of *Doratoxylon apetalum*, a medicinal plant that is common in the Mascarene Islands and rich in polyphenols, showed activity against ZIKV, targeting the initial stages of infection. In that study, it was proposed that the extract might bind to viral particles to inhibit ZIKV internalization. Thus, it was not possible to determine which compounds were responsible for the loss of viral infectivity, an issue that still needs to be investigated (Haddad et al., 2019).

Despite presenting potential for the discovery of new molecules/activities, antiviral research using unfractionated plant extracts presents some limitations, such as the possibility that the composition of the secondary metabolites may vary depending on the soil characteristics, climate, radiation, and period of plant collection (Patil et al., 1995; Van der Sluis et al., 2001). It is also essential to identify bioactive compounds, which represents a challenge for researchers working on the purification and characterization of compounds. These are common issues that should be considered when studying the biological/pharmacological properties of plant extracts. Below, we discuss the current data on the anti-ZIKV effects of isolated flavonoids.

##### Sophoraflavenone G

*Sophora flavescens* is a medicinal herb with a long history in Asian countries that is composed of hundreds of compounds, including a variety of flavonoids derived from the roots. The roots of this herb have been used to treat fever, pain, and several other symptoms (He et al., 2015). Sze et al. (2017) identified sophoraflavenone G derived from the roots of *S. flavescens* as an antiviral compound against DENV and ZIKV. The isolated molecule, sophoraflavenone G (5,7,2',4'-tetrahydroxy-8-lavandulylflavanone), is a flavanone analog of naringenin with two stereogenic centers, bearing an additional hydroxyl substituent at the 2' position and a (2R)-5-methyl-2-(prop-1-en-2-yl)hex-4-en-1-yl (lavandulyl) substituent at the 8' position. Sophoraflavenone G treatment after infection led to a reduction in the frequency of DENV- and ZIKV-infected cells. These data suggest that sophoraflavenone G affects the later steps of the virus replication cycle (Figure 3). To address the anti-ZIKV mechanism of the compound, the authors performed an RNA-dependent RNA-polymerase (RdRP) activity assay and observed the inhibition of this enzyme. Despite the IC_50_ of 22.61μM and the low selectivity index (SI) of sophoraflavenone G, the specific inhibition of ZIKV-RdRP has the potential to cause fewer side effects *in vivo* (Sze et al., 2017). However, the *in vivo* efficacy of sophoraflavenone G against ZIKV needs to be determined.

##### Baicalein and Baicalin

*Scutellaria baicalensis* is a Chinese medicinal herb that presents a high concentration of the flavonoid baicalein in its roots, and its glucoside form, baicalin, is the most abundant glucoside flavonoid that has been isolated. Baicalein, 5,6,7-trihydroxy-2-phenyl-chromen-4-one, is a trihydroxyflavone with hydroxyl groups at positions C-5, C-6, and C-7. Its derivative baicalin is a flavone glycoside with a glucuronide moiety at the 7-OH position (Table 1). The low bioavailability of baicalin could limit its use and compromise its clinical efficacy (Huang et al., 2019). Due to its high polarity, transportation is limited through the lipid bilayer *via* simple diffusion (Huang et al., 2019). Baicalein presents good permeability due to its good lipophilicity. Thus, changes in the structure of baicalin that enhance its bioavailability would be beneficial to potentiate its biological activity.

Baicalein exerts dual antiviral activity against DENV through its virucidal activity and by inhibiting virus adsorption and replication (Zandi et al., 2012). The anti-ZIKV activity of baicalein and baicalin was assessed by Oo et al. (2019). Using time-course experiments, a 50% reduction in ZIKV RNA levels in cell culture supernatants was observed for both baicalein- and baicalin-treated Vero cells. Both compounds were able to impair the infection during different steps of the viral cycle. However, baicalein was shown to be more potent when added after infection, while baicalin seemed to be most effective in the early stages of the viral cycle (Figure 3). Due to their low toxicity, baicalin could be promising in the treatment of ZIKV infection due to its high affinity for the NS5 protein (RdRP), corroborated by docking analysis (Oo et al., 2019). Additionally, as ZIKV NS5 inhibits STAT2 (Grant et al., 2016), the binding of baicalin to NS5 could modulate the innate immune response to better control virus replication due to type I IFN production.

##### Epigallocatechin Gallate

Epigallocatechin gallate is the main natural flavonoid found in green tea and exerts antiviral activity against herpes simplex virus, hepatitis C virus, and DENV (Lyu et al., 2005; Ciesek et al., 2011; Raekiansyah et al., 2018). EGCG comprises a catechin group, similar to epicatechin, with a benzopyran moiety similar to catechol and two stereocenters with the relative and absolute stereochemistry of cis and 2R,3R, respectively. Its synthesis was reported for the first time by Li and Chan (2001).

To assess the antiviral activity of EGCG against ZIKV, Carneiro et al. (2016) preincubated viral particles for 1h with different concentrations of EGCG and then inoculated Vero E6 cells with the mixtures (Carneiro et al., 2016). A 1-log reduction in the number of viral foci was observed in the cell cultures. African lineage ZIKV seemed to be more susceptible to EGCG activity, as lower concentrations of the compound were needed to decrease the foci number to the same extent as that of the Brazilian isolate of Asian lineage. These results suggested a direct action of the drug in the viral particle, possibly by the interaction with the phospholipid envelope, which could inhibit viral adsorption onto the cell surface and impair viral entry into the cells. Molecular dynamics simulations and docking studies showed that EGCG blocked the fold back of domain III, which is an important step in forming the hairpin structure (Sharma et al., 2017). Therefore, the interaction between EGCG and ZIKV E protein blocks viral host membrane fusion, impairing viral RNA release into the cytoplasm. Additionally, it was recently shown that EGCG presents a strong interaction with the ATPase region of the ZIKV-NS3 protein with a binding energy of 7.8kcal/mol (IC_50_ of 295.7μM; Kumar et al., 2020). Vázquez-Calvo et al. (2017) obtained similar results when testing EGCG against ZIKV, DENV, and *West Nile virus* (WNV; Vázquez-Calvo et al., 2017). In contrast, Raekiansyah et al. (2018) observed minimal effects from EGCG against ZIKV, despite the high activity against the four serotypes of DENV. These opposite results may be explained by differences in the viral strains used, which reinforces the need to test as many virus isolates/strains and cells as possible (Raekiansyah et al., 2018).

##### Quercetin-3-β-O-D-Glucoside (Q3G or Isoquercitrin)

The anti-ZIKV activity of quercetin-3-β-O-D-glucoside (Q3G) was recently evaluated. Q3G is also known as isoquercitrin and is one of the main glycosidic forms derived from the flavonoid quercetin. It is noteworthy that glycoside forms of quercetin are rapidly absorbed and exhibit higher bioavailability than their corresponding aglycone forms (Arts et al., 2004), which are important features for antiviral candidates. The anti-ZIKV activity of Q3G was first tested *in vitro* in Vero cells. Q3G treatment before and after ZIKV infection showed a reduction in NS1 RNA (using RT-qPCR) in the supernatant and cell lysate at the 2nd and 4th days post-infection, suggesting an effect on viral replication. Additionally, *in vivo* assays in immunocompromised mice (type I IFN receptor knockout – IFNAR1^−/−^) demonstrated 50% survival up to 21days after ZIKV infection upon Q3G treatment, which contrasted with 100% death within 7days of infection in the vehicle control group (Wong et al., 2017).

Gaudry et al. (2018) also showed that the addition of Q3G (100μM) at the beginning of infection was able to decrease the number of A549-infected cells, intracellular viral RNA, and viral titers in the supernatant (Gaudry et al., 2018). The antiviral effects of Q3G were shown by an internalization assay, suggesting that Q3G may act as an inhibitor of virus internalization. Furthermore, the same study also evaluated the related Q3G flavonoids quercetin, hyperoside, and kaempferol, but no anti-ZIKV effects were observed for these compounds (Gaudry et al., 2018).

##### Phloretin

The anti-ZIKV activity of phloretin was tested *in vitro* against infection with both African and Asian lineages. Phloretin treatment was able to decrease the cell death rate induced by ZIKV infection, viral progeny, and intracellular ZIKV envelope protein staining. Interestingly, the antiviral effects of phloretin seem to be cell-type dependent since higher activity was observed in Vero and U87MG cells than in human umbilical vein endothelial cells (HUVECs). In a time-course assay, the results showed that phloretin was able to interfere in multiple steps of the viral cycle, but mainly in RNA production and later steps of the viral cycle (Figure 3; Lin et al., 2019). Moreover, the importance of glucose for ZIKV replication was demonstrated, and phloretin induced a significant reduction in viral titers in cells cultured in glucose-free medium (Lin et al., 2019). Phloretin is a glucose transporter (GLUT1) inhibitor, and the ZIKV effect on GLUT1 has been hypothesized to be involved in the risk of congenital syndrome (Blonz, 2016). The AKT/mTOR pathway has been demonstrated to be involved in the increase in glucose uptake and metabolism (Kohn et al., 1996). In addition, mutations of viral proteins such as NS4A and NS4B have been reported to induce the inhibition of the AKT/mTOR pathway, which is important in the development of neuropathies (Jun et al., 2017). Lin et al. (2019) also investigated the AKT/mTOR pathway and observed decreased phosphorylation of the AKT/mTOR pathway in infected phloretin-treated cells, suggesting a possible involvement of this pathway in virus replication (Lin et al., 2019). Altogether, these data suggest a positive correlation between GLUT1 transporter expression and ZIKV titers. In agreement with these data, it was demonstrated that ZIKV infection can upregulate glycolysis metabolism to support replication (Tiwari et al., 2017). Moreover, phloretin was revealed to exert *in vitro* neuroprotective effects in human neuroblastoma cells (Barreca et al., 2017). Therefore, phloretin would be an interesting molecule for further studies since it impairs ZIKV replication and shows neuroprotective ability. However, it is important to determine the relationship between glucose metabolism and viral replication before considering this compound as an antiviral drug, mainly in the context of pregnancy and fetal development.

##### Pinocembrin

Pinocembrin (5,7-dihydroxyflavanone) is a chiral flavanone isolated from a variety of plants, mainly from Pinus heartwood, *Eucalyptus* spp., *Populus* spp., *Euphorbia* spp., and *Sparattosperma leucanthum*. It is also a compound that is widely distributed in honey and propolis with neuroprotective characteristics (Guang and Du, 2006). It is a major flavonoid with industrial applications since it has several pharmacological activities (Rasul et al., 2013).

Following an immunofluorescence-based high-throughput screening assay of 483 flavonoid derivates, Lee et al. (2019) identified the flavanone pinocembrin to have a high inhibitory effect on ZIKV, with an IC_50_ of 17.4μM. The anti-ZIKV activity of pinocembrin was observed to be cell type-dependent, inhibiting infection of Huh7 (human-derived hepatoma) cells; however, pinocembrin presented no reduction in infection in the BHK-21 and HEK293T (human embryonic kidney) cell lines. Additionally, pinocembrin seems to interfere with viral RNA synthesis and envelope proteins, directly affecting viral replication (Lee et al., 2019). Interestingly, pinocembrin was already approved in China for phase II clinical trials in ischemic stroke patients (U.S. National Institutes of Health, n.d.), making its repurposing as an anti-ZIKV compound an attractive alternative.

##### Naringenin

Naringenin (NAR; 4,5,7-trihydroxyflavanone) is the natural flavonoid aglycone of naringin with one asymmetric center. Regarding its anti-flavivirus effects, NAR exerts antiviral activity against all four serotypes of DENV and against Asian- and African-lineage ZIKV (Frabasile et al., 2017; Cataneo et al., 2019). A549 cells infected by both lineages of ZIKV showed decreases in the rate of infected cells, the amount of viral RNA inside the cells, and the production of viral progeny in the culture supernatants after treatment with NAR (IC_50_ 58.79μM). Furthermore, a set of drug addition time-course experiments suggested that the antiviral activity of NAR should occur at later steps of the virus cycle, between replication and viral release events. Molecular docking studies have shown interactions between NAR and the protease domain of the NS2B-NS3 protein. NAR interacts *via* hydrogen bonds with residue Gln-74 in the viral protein, the carbonyl group of NAR acts as a hydrogen acceptor of the acid portion of the carboxylic acid of residue Gln-167, and the 6- and 7-hydroxyl phenolic moieties of NAR become hydrogen bond donators to the Thr-166 and Gln-167 carboxyl groups, respectively. The oxygen at the 4’-OH position bonds to the amine hydrogen of Trp-89. In addition, hydrophobic interactions occur through contact of the Ile-123 side chain with NAR carbon atoms (Cataneo et al., 2019). Therefore, docking studies reinforced the hypothesis of viral replication inhibition by NAR. Interestingly, while Frabasile et al. (2017) observed that NAR impairs DENV replication with no virucidal effects (Frabasile et al., 2017), Zandi et al. (2011) showed a virucidal effect and that NAR was unable to block DENV replication (Zandi et al., 2011). These contrasting results might be explained by differences in the cell type and/or DENV strains used in each set of experiments. Additionally, *in vivo* assays in small animal models should be performed to confirm the potential of NAR to treat ZIKV-infected patients.

#### Structure: Activity Relationship

The data presented herein suggest that flavonoids could be a source of suitable molecules to fight ZIKV infection. The inhibitory activity of flavonoids impacts different steps of ZIKV morphogenesis in host cells, such as binding and entry, viral replication, maturation and release, and the host antiviral immune response. These mechanisms of action may contribute to controlling the infection and/or reducing damage to the host.

The antiviral activity of compounds depends mainly on the interaction between an active site of a molecule and a virus or cellular protein, which depends on intermolecular forces such as hydrophilicity, hydrophobicity, electrostatics, and sterics. Numerous substituents can be found on the rings of the flavonoid structure, such as hydroxyl, methoxy, benzyl, and methyl groups, and as mentioned above, flavonoids can also undergo glycosylation at different positions (Feng et al., 2017). Additionally, chemically directed changes to the structures of flavonoids could be rationally used to improve activity, reduce toxicity, and increase the half-life of the compounds. Although, flavonoids are potential candidate antimicrobial drugs, they present low bioavailability. To improve their biological activities and pharmacokinetic properties, structural modifications could be a good strategy (Thilakarathna and Vasantha Rupasinghe, 2013). By introducing nonpolar groups, such as hydrocarbon chains, their solubility in lipid phases could be improved, such as etherifying ring A phenols with groups such as methoxy or other alkyl chains. Additionally, bioavailability could be improved by increasing the number of polyhydroxylated groups, which are responsible for the solubility enhancement in biological environments *via* hydrogen bonding with water (Chimento et al., 2019).

Flavonoid activity relies mainly on the phenol group, which, due to its high acidity, becomes a nucleophilic species with weak bases, allowing the insertion of alkyl, alkynyl, triazole, and several other functional groups. The addition of alkyl chains is achieved through an SN2 reaction with alkyl halides. The 1,2,3-triazole-bridged flavonoids can be accessed through an alkynyl ether *via* click chemistry reactions with azide functionalization (Sum et al., 2016). Additionally, Zou et al. (2020) demonstrated that the presence of hydroxyl groups in the B-ring is critical for the antiviral activity of flavonoids (Zou et al., 2020).

Suroengrit et al. (2017) demonstrated the anti-ZIKV and anti-DENV activities of halogenated derivatives of chrysin. The electronegative substituents bromine and iodine at positions 6 and 8, in addition to the free hydroxyl groups at positions 5 and 7, contributed to the antiviral activities of these molecules. These compounds were able to inhibit virus production by up to 62% at a concentration of 10μM, while the halogenated derivatives showed inhibitory activity greater than 99% at the same concentration. The halogenated derivatives showed potent activity *in vitro* against all serotypes of DENV and ZIKV and a similar low cytotoxicity, suggesting that these compounds are broad-spectrum anti-flavivirus drugs with activity against multiple targets; however, the greatest efficacy was achieved with early posttreatment (Suroengrit et al., 2017).

A study conducted by Du et al. (2016) demonstrated the antiviral activity of a chrysin analog, a phosphate ester substituted at position 7 by a diisopropyl phosphate group, against DENV1 and DENV2. The phosphorylated analog inhibited viral protein synthesis in cells infected with DENV1 and DENV2, with IC_50_ values of 18.6 and 15.1μmol L^−1^, respectively, demonstrating efficacy against the replication of these two serotypes in cell culture without detectable cytotoxicity (Du et al., 2016). Chen et al. (2004) demonstrated that phosphorylated flavonoids have strong binding affinities in addition to forming noncovalent complexes with many proteins more easily than nonphosphorylated compounds (Chen et al., 2004).

Motivated by the biospherical relationship between quercetin and aryl diketoacid (ADK), known for its antiviral activity, in addition to the role of arylmethyl substituents in antiviral activity, Park et al. (2012) reported the synthesis and antiviral evaluation of 7-O-benzylated quercetin derivatives (7-O-arylmethylquercetin) against hepatitis C (HCV) and SARS-associated coronavirus (SARS-CoV, SCV). Concerning the anti-HCV activity, among the synthesized compounds, the derivatives containing strong electron-withdrawing groups in the meta and para positions of the benzyl ring, such as a nitro group, proved to be the most active without showing cytotoxicity. In addition, the presence of electron-donating groups, such as hydrogen, methyl, and methoxy groups, at position four (4″) of the benzyl ring also showed selective activity. These data suggest that the electronic properties of these groups around the benzyl ring play an important role in the bioactivity of these molecules (Park et al., 2012).

Lim et al. (2017) demonstrated the inhibitory activity of 22 selected flavonoids against ZIKV NS2B-NS3. It was shown that the number and position of hydroxyl groups favor the anti-ZIKV activity of flavonoids, while glycosylation, methoxy, and prenyl groups seem to exert lower inhibitory effects on ZIKV NS2B-NS3 (Lim et al., 2017). Thus, although glycosylation increases the absorption of flavonoids, it decreases the inhibitory efficacy against ZIKV. Moreover, the presence of oxygen within a ring seems to be crucial for inhibitory activity. Conversely, glycone moieties are detrimental to inhibitory activity (Bhargava et al., 2017). Therefore, the Monte Carlo-based QSAR model could be a suitable tool for the prediction of new antiviral compounds, helping to identify active molecules in a short time frame, which is essential for preparedness policies related to emergency global public health concerns, such as what has been witnessed for ZIKV and influenza outbreaks and more recently for SARS-CoV-2.

Recently, changes in the NAR molecule were made to improve its bioavailability and anti-ZIKV activity. Naringenin-derived mono-7-O-ether and mono- and di-fatty acid ester compounds were synthesized and tested *in vitro* for their anti-ZIKV activity. Lipophilic ether derivatives of naringenin presented anti-ZIKV activity but also showed higher cytotoxicity than unmodified NAR without any improvement in the selectivity index (Albuquerque de Oliveira Mendes et al., 2020).

A vast body of literature describing flavonoids as biological molecules is available. Nevertheless, the biological mechanisms of action of flavonoid-based compounds against ZIKV have not been deeply explored. Therefore, chemical modifications targeting flavonoid structures represent a rational approach to improve anti-ZIKV activity, optimize pharmacokinetics, and reduce the toxicity of potential anti-ZIKV flavonoids.

## Concluding Remarks

The potential use of flavonoids as a source of molecules with anti-ZIKV activity was addressed in this review. Although promising, several questions remain to be answered. As flavonoids have become an important class of compounds with potential biological activities, it is essential that these molecules are well absorbed by the gastrointestinal tract and present minimal side effects. *In vitro* cell culture models have been used to simulate the ability of flavonoids to permeate the intestinal tract, and hydrophobic molecules were found to have better absorption potential (Barrington et al., 2009). The absorption and bioavailability of flavonoids can vary depending on different factors. As stated before, the flavonoid chemical structure is an important feature that determines their biological activity but also impacts the *in vivo* absorption and bioavailability.

Some of the data reviewed herein demonstrate that glucoside forms of flavonoids have better absorption (Dai et al., 2008). In general, several mechanisms are involved in the impaired bioavailability of flavonoids, such as poor transport due to glycoside characteristics or enhanced metabolism of the aglycones, leading to low concentrations available to exert the desired biological effect. However, some strategies can be adopted to overcome this barrier, such as improving intestinal absorption with microemulsions and nanocarriers (Constantinides, 1995; Lembo et al., 2018) or structural modifications leading to enhanced hydrophobicity, improving the delivery and efficacy of the molecules. Indeed, the absorption, metabolism, transport, and bioavailability of flavonoids are great challenges to overcome in the drug discovery field. Advances in this field are essential to allow the progress of *in vitro* bioactive compounds to preclinical tests in animal models and eventually clinical tests in humans.

The identified active compounds against ZIKV were mostly evaluated by *in silico* or *in vitro* studies using computational and cell-based assays and need to be further evaluated in preclinical assays using small animal models before redirection to clinical tests in humans. Preclinical assays using small animal models are a special challenge regarding flavivirus infection. Most murine models of flavivirus infection lack important components of the immune response (Dowall et al., 2016; Lazear et al., 2016). Despite the limitations of *in vivo* flavivirus infection, susceptible animals presenting quantifiable viremia and measurable clinical scores can be a suitable model to study the efficacy of antiviral molecules and the pharmacokinetic and toxicological effects of flavonoids.

In addition to evidence that supports flavonoids as promising compounds to exert antiviral activity, several challenges persist. The lack of a ZIKV-immunocompetent animal model, flavonoid purification, structure-activity relationships, toxicity and pharmacokinetic properties of the molecules, and determination of their precise mechanism of action need to be fully addressed. Finally, flavonoids are present in our diet and are also taken as supplements; nevertheless, all the questions mentioned above must be considered before a flavonoid-based anti-ZIKV molecule proceeds to clinical trials. Regardless, the data reported to date support the potential therapeutic applicability of flavonoids against ZIKV.

## Author Contributions

AC, JB, and PW: conception, data curation, and drafting the manuscript. EA, LM, VO, CF, MA, SF, CD, and WV: data curation and drafting the manuscript. MA, SF, CD, WV, JB, and PW: critical revision of the article. All authors contributed to the article and approved the submitted version.

## Funding

This research was funded by the Brazilian Ministry of Health and the Fundação Araucária for their financial support (PPSUS/2016) and Fundação Araucária/SESA-PR/CNPq/MS-Decit PPSUS/2015 – projects 009/2017, 019/2017 and 041/2017, CNPq PROEP (442322/2019-4), CNPq (427946/2018-2), and PRONEX-Fundação Araucária/SETI/MCTIC/CNPq/Paraná State Government (agreement 014/2017, protocol 46.843). CD (307176/2018-5), WV (307186/2017-2), and JB (303306/2017-3) are CNPq fellows.

## Conflict of Interest

The authors declare that the research was conducted in the absence of any commercial or financial relationships that could be construed as a potential conflict of interest.

## Publisher’s Note

All claims expressed in this article are solely those of the authors and do not necessarily represent those of their affiliated organizations, or those of the publisher, the editors and the reviewers. Any product that may be evaluated in this article, or claim that may be made by its manufacturer, is not guaranteed or endorsed by the publisher.

## Footnotes

^1^ https://www.worldcommunitygrid.org/
